# Items

**Published:** 1892-01

**Authors:** 


### ATLANTA SOCIETY OF MEDICINE.

---

At the December meeting of the Society officers for the following year were elected. The retiring President, Dr. McRae, delivered his final address in which he reviewed the work done by the Society during his administration. For the present year the officers will be: President, Dr. W. S. Kendrick ; Vice-President, Dr. J. A. Childs ; Secretary, Dr. C. D. Roy ; Librarian, Dr. J. D. Wilson ; Reporters, Dr. R. R. Kime, Dr. L. B. Grandy and Dr. H. F. Harris.

---

The friends of Dr. J. McFadden Gaston are pleased at his having received the well-bestowed honor of being elected President for the ensuing year, of the Southern Surgical and Gynecological Association at its recent meeting in Richmond, Va.

---

The best remedy to loosen expectoration is muriate of ammonium, combined with some such remedy as syrup of squills. If dry plastic pleurisy be present, iodide of ammonium should be added as follows :

℞.—Ammonii muriat....................ʒiiss.

Syr. scillæ..........................℥ij.

Ammonii iodidi......................ʒiss.

Glycerinæ...........................℥ss.

Syr. prun. Virg...............q. s. ad. ℥iv.

M.—Sig. ʒj every three hours in water.

At the same time in a case of pleurisy, to prevent any possible tubercular tendency, give something to build up tissue and aid general strength; something to strengthen the chest. The best thing for this purpose is the syrup of the hypophosphites, ʒi three times a day after meals.—*Anders.—Ex.*

